# Small Clinical Charts

**Published:** 1892-03

**Authors:** 


*Small Clinical Charts.* London: H. K. Lewis.

These clinical charts are modelled on the ordinary type, and the temperature may be registered by them either in degrees Fahrenheit or Centigrade. They are very much smaller than those commonly in use, and may thus be found more convenient for special uses. Though small, they are very clearly printed.

